# Slow Invaders Going Fast: New Data of Exotic Slugs (Gastropoda: Eupulmonata) From Spain

**DOI:** 10.1002/ece3.71306

**Published:** 2025-05-14

**Authors:** Omar Sánchez, Víctor González‐García, Jairo Robla, Andrés Arias

**Affiliations:** ^1^ Department of Biology of Organisms and Systems (Zoology) University of Oviedo Oviedo Spain; ^2^ Biodiversity Research Institute—IMIB (University of Oviedo – CSIC‐Principado de Asturias) Mieres Spain; ^3^ Department of Conservation Biology & Global Change Doñana Biological Station—CSIC Sevilla Spain; ^4^ Institute of Natural Resources and Territorial Planning (INDUROT) University of Oviedo Mieres Spain

**Keywords:** *Ambigolimax parvipenis*, anatomical study, *Boettgerilla pallens*, introduced species, molecular characterisation, synanthropic slugs

## Abstract

Invasive species are a major concern in the current scenario of biodiversity loss. Most studies focus on vertebrates and insects, while other groups have been profoundly overlooked. Particularly, terrestrial slugs are among the most understudied taxa. Here, we contribute to expanding the scarce knowledge on exotic invertebrates by reporting the occurrence of two non‐native terrestrial slugs in the Iberian Peninsula (northern Spain): *Ambigolimax parvipenis,* being also the first morphological confirmation for continental Spain, and *Boettgerilla pallens*, constituting the first record for continental Spain as well. Both species were collected in gardened urban areas or in peri‐urban areas with a high presence of exotic ornamental plants. Some individuals were used for anatomical studies, while other ones were subjected to DNA extraction and PCR amplification and sequencing, comparing their sequences with closely related species from GenBank and confirming their previous morphological identification. Early detection of exotic species is crucial to avoid potential future threats. Unfortunately, many non‐native species remain understudied or misidentified, leading to a silent invasion. Our findings entail a new step in the understanding of this neglected group and in the implications of urban gardened areas as a source of arrival of exotic fauna.

## Graphical abstract

Morphological and genetical confirmation of the occurrence of two non‐native terrestrial slugs in the Iberian Peninsula (northern Spain): Ambigolimax parvipenis and *Boettgerilla pallens*.

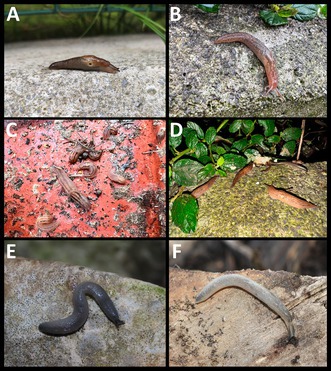

## 1 Introduction

Globalisation has facilitated both the deliberate and accidental introduction of species beyond their natural habitats across the globe (Hulme 2009; Turbelin et al. 2022). Currently, invasive species are considered one of the most significant global challenges to biodiversity (Crystal‐Ornelas and Lockwood 2020). They have profound ecological consequences (Linders et al. 2020), displacing native (mainly endangered) species and altering ecosystem dynamics (Duenas et al. 2021; Wainright et al. 2021; Fortuna et al. 2022). Furthermore, they are a major cause of economic losses (Epanchin‐Niell 2017; Warziniack et al. 2021). Additionally, certain invasive species pose medical concerns, particularly when they carry pathogens that can affect livestock or humans (Pejchar and Mooney 2009; Chinchio et al. 2020; Schmeller et al. 2020; Sánchez et al. 2021) or may become significant agricultural and forestry pests (Holmes et al. 2009; Paini et al. 2016). Given their potential impact, it is crucial to address various aspects of invasive species management.

From a management perspective, different stages can be identified, from the arrival of a species to its complete establishment (Hulme 2006; Robertson et al. 2020). First, the most crucial step is preventing the arrival of the alien species (Leung et al. 2002; Kim et al. 2006). If the species arrives, rapid detection and an early response for eradication are essential to avoid potential future threats (Reaser et al. 2020). If the species spreads, management moves into the control, monitoring, or containment phase, where it is important to limit the expansion or eradicate the invasive species (e.g., Bloem et al. 2013). Finally, if it is no longer possible to limit the invasion, the final step is the restoration and mitigation of its impacts (Perrings 2005; Guo et al. 2018). Effective prevention or rapid detection often requires a comprehensive understanding of the species in question. However, many non‐native species are poorly known or inadequately characterized, lacking proper anatomical, morphological, ecological, or genetic differentiation from native fauna (Miglietta and Lessios 2009; Morais and Reichard 2018; Sánchez et al. 2024). This gap in knowledge hampers the effectiveness of invasion management. As a result, species may enter, establish and spread silently without being detected for a long time (Janssen 2019; Zogaris et al. 2023), or may be noticed but misidentified. During this period, the species can cause damage to native ecosystems without being recognized as introduced or invasive (Marble and Brown 2021). Furthermore, studying the pathways of introduction is important to determine where preventive measures should be implemented to curb the arrival of invasive species (Faulkner et al. 2020; Turbelin et al. 2022). Lastly, monitoring and mapping the distribution of introduced species is also critical to assess their current geographic range (Wallace and Bargeron 2014) and the future one (Barbet‐Massin et al. 2018).

Among invasive species, vertebrates have garnered particular attention, with several well‐known examples (Pitt and Witmer 2014; Lockwood et al. 2019). However, invertebrates also have a significant history of invasions, being insects (Fortuna et al. 2022) and both terrestrial and aquatic molluscs (Steger et al. 2022; Darrigran et al. 2023; Mahapatra et al. 2023) some of the most studied groups due to their high impact. Specifically, terrestrial slugs (Mollusca: Gastropoda) are often overlooked and understudied. Land slugs include numerous invasive species (Vendetti et al. 2018), which are often difficult to distinguish from native fauna. Some species are capable of displacing native slug fauna, leading to ecological simplifications in invaded ecosystems (Zemanova et al. 2017). Most slug species feed on plants, including vegetables, fruits, mushrooms and ornamental flowers, and can become serious agricultural pests, potentially destroying crops (Kozłowski and Kozłowski 2011; Zajac et al. 2017), or even affect native plant communities (Joe and Daehler 2008; Hahn et al. 2011). Furthermore, many species carry parasites that may invade new ecosystems, infecting plants, livestock and even humans (Ross et al. 2010; Sánchez et al. 2021).

Many slug species are considered introduced outside their natural habitats (e.g., Reise et al. 2000; Vendetti et al. 2018), as they can easily spread through the movement of horticultural or ornamental plants (Cowie et al. 2008). Historical human activity has obscured the native range of many species, and due to their morphological similarity to native species, some introduced slugs have remained undetected for long periods. This makes studying introduced slugs a challenging task. For example, three introduced species are currently known in Spain. One case is *Deroceras invadens* Reise et al. 2011, a species that was long historically confused with *Deroceras panormitanum* (Lessona & Pollonera, 1882) and has only recently been properly characterized (Reise et al. 2011; Hutchinson et al. 2014, 2020). Another classic example is the so‐called Spanish slug, *Arion vulgaris* Moquin‐Tandon, 1855, historically misperceived as *Arion lusitanicus* (Pfenninger et al. 2014; Zajac et al. 2017), making it difficult to track its distribution and invasion, and their native or introduced areas. The third case is *Ambigolimax parvipenis* Hutchinson et al. 2022, considered an invasive species with an uncertain and unknown origin (Hutchinson et al. 2022; Hart et al. 2023). These three cases are examples where the original distribution area, invasion history, or differentiation from native species is unclear, highlighting the need for further morphological, ecological and genetic studies on land slugs.

Therefore, the objectives of this study are to (a) present a morphological and genetic characterisation of two previously unreported introduced slug species in northern Spain; (b) provide ecological and biological remarks of these new populations; and (c) discuss potential pathways of introduction and impacts.

## 2 Material and Methods

### 2.1 Specimen Collection and Morphological Analysis

**FIGURE 1:**
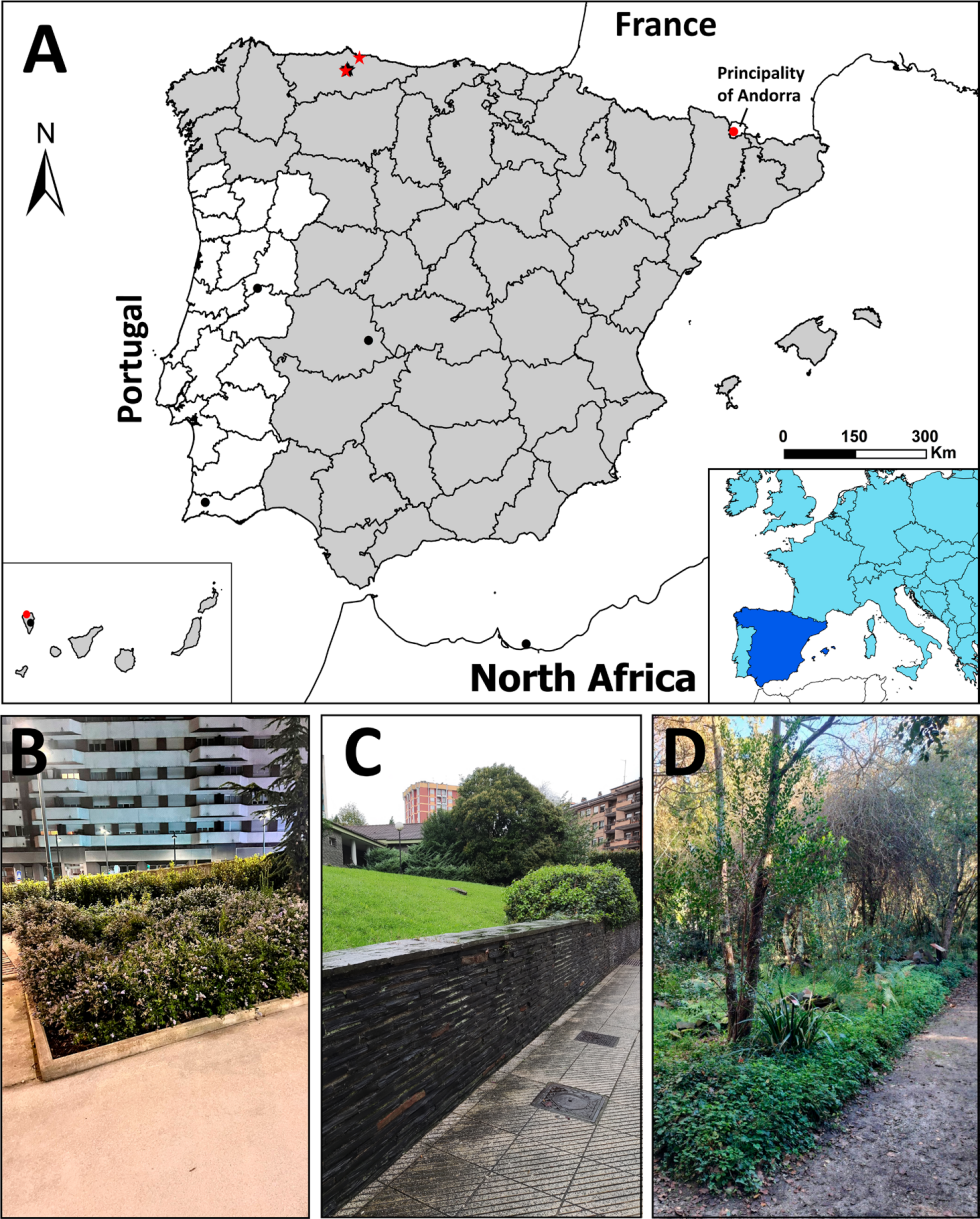
Distribution of known records of *Ambigolimax parvipenis* (black) and *Boettgerilla pallens* (red) in the Ibero‐Balearic region and Canary Islands. Records are from bibliography (circles) and from our own samples (stars). Points in proximity may appear to overlap. Spain is marked in light grey (A). (B–D) Places where specimens were located. (B) Surroundings of «Plaza de la Poesía» at the Gran Bulevar ‘El Vasco’ shopping center (Oviedo); (C) Garden of the «El Cristo» medical center (Oviedo) and (D) Atlantic botanical garden (Gijón), ‘Cantabrian forests area’.

A total of 76 specimens of *Ambigolimax parvipenis* and nine of *Boettgerilla pallens* Simroth 1912 were collected between 2022 and 2024 from several locations in the Northern Iberian Peninsula, specifically Asturias (Spain) (Figure 1A). Specimens were hand‐collected in urban or suburban anthropized gardened areas, primarily under logs, rocks and among leaf litter. For anatomical studies, all of these specimens were subjected to a relaxation process by immersing them in hot water with menthol crystals for 24 h and then preserved in 70% ethanol. Before being fixed, a piece of sole tissue was removed and stored in 96% ethanol for molecular analyses. The specimens were deposited in the National Museum of Natural Science (MNCN; Madrid, Spain) and in the personal collection of the first author (OSF; Asturias, Spain). Slugs were examined and dissected using an Optika SZM‐2 stereomicroscope (0.7–4.5X). Photographs of live molluscs were taken with a Canon EOS 1200D digital SLR camera equipped with an EF‐S 18–55 mm f/3.5–5.6 III lens. Maps were generated with ArcGis ArcMap 10.6. The botanical characterisation of the study areas was made following the keys of ‘Flora Iberica’ (Castroviejo 1986–2012) and ‘Illustrated keys to the flora of the Basque Country and neighbouring territories’ (Aizpuru et al. 1999), following the taxonomy of ‘Plants of the World Online’ (POWO 2024). For examined material, abbreviations are as follows: OSF *col*. = Omar Sánchez Fernández personal collection (Asturias, Spain), MNCN *col*. = National Museum of Natural Science collection (Madrid, Spain), *ex*. = specimen, *deposit*. = collection of deposit and *leg*. = collector.

### 2.2 DNA Extraction, PCR Amplification and Sequencing

Six specimens of *A. parvipenis* and one of *B. pallens* were subjected to the DNA extraction process to confirm the previous morphological identification. The DNA was extracted from 30 mg of ethanol‐preserved tissues from the sole, using the E.Z.N.A. Mollusc Kit (Omega Bio‐tek). DNA samples were stored at −20°C. The mitochondrial cytochrome c‐oxidase subunit I (COI) gene fragment was amplified by Polymerase Chain Reaction (PCR) in a total volume of 30 μL, using LCO1490 (Folmer et al. 1994) and COR722 (Davis et al. 1998) as primers. PCR reaction mixture and conditions followed Martín‐Álvarez et al. (2024). Horizontal electrophoresis (1% agarose gel) with 0.05 μL/mL of SimplySafe (EURx Ltd. 80–297 Gdańsk Poland) was performed. Finally, forward and reverse sequencing was performed at MACROGEN (Madrid, Spain), using the standard Sanger sequencing method (Sanger and Coulson 1975).

### 2.3 Genetic Analysis

The forward and reverse sequences obtained by Sanger sequencing were edited using Geneious Prime 2024.0 (https://www.geneious.com) for quality trimming and primer removal. Then, they were aligned and manually checked to correct any possible wrong base calling. A genetic species identification was attempted using nBlast implemented in Geneious Prime using the default values to search in GenBank databases. For constructing the phylogenetic trees, the six novel sequences of *A. parvipenis* (Accession Numbers: PQ327557–PQ327558; PQ563291–PQ563294) were aligned with a set of 61 previously published sequences deposited in GenBank of different slug species from the Limacidae family, among them *A. valentianus* (Férussac, 1821), *A. parvipenis*, *Lehmannia marginata* (O. F. Müller, 1774), *Malacolimax tenellus* (O. F. Müller, 1774), *Limax maximus* Linnaeus, 1758, *Li. cinereoniger* Wolf, 1803, and one sequence of a slug of the Arionidae family, namely *Arion intermedius* (Normand, 1852), which was used as an outgroup. On the other hand, the new sequence of *B. pallens* (Accession Number: PQ327549) was aligned with a set of 27 previously published sequences deposited in GenBank, belonging to the family Boettgerillidae with *B. pallens* and *B. compressa* (Simroth 1910), and other species from different families, like *Deroceras invadens*, *De. laeve* (O. F. Müller, 1774), *Milax gagates* (Draparnaud, 1801), and one sequence of *Ar. Intermedius*, as outgroup. The list of accession numbers can be checked in Appendix S1. Phylogenetic analyses were conducted using IQ‐TREE v2.3.1 software (Minh et al. 2020). The ModelFinder option included in IQ‐TREE was used to predict the nucleotide substitution model showing the best BIC scores (Kalyaanamoorthy et al. 2017). A Maximum Likelihood tree was performed using Ultrafast Bootstrap options (1000 bootstrap replicates) (Minh et al. 2013), and a search was conducted for the best‐scoring tree using the TPM2u + F + I + G4 model. Moreover, the PopART 1.7 program using the Median‐Joining Model (Bandelt et al. 1999) was used for obtaining the haplotype networks.

## 3 Results

### 3.1 Taxonomy

Phylum Mollusca Linnaeus, 1758.

Class Gastropoda Cuvier, 1795.

Order Stylommatophora A. Schmidt, 1855.

Superfamily Limacoidea Batsch, 1789.

Family Limacidae Batsch, 1789.

Subfamily Limacinae Batsch, 1789.

Genus *Ambigolimax* Pollonera, 1887.

*Ambigolimax parvipenis* (Hutchinson et al. 2022).

Material examined. SPAIN: Principality of Asturias: Oviedo: surroundings of «Plaza de la Poesía» at the Gran Bulevar ‘El Vasco’ shopping center: 2 *ex*. adults and 54 subadults (A. Arias *leg*. and OSF *deposit*.), 24‐IV‐2024, gardened urban area (30T 269,720 4,805,339); 4 *ex*. adults (A. Arias *leg*. and 2. *ex*. MNCN 15.05/98400 + 2. *ex*. OSF *deposit*.), 09‐VII‐2024, same location; 8 *ex*. adults (A. Arias *leg*. and 6. *ex*. MNCN 15.05/98403 + 2. *ex*. OSF *deposit*.), 16‐X‐2024, same location; Oviedo: Pista Finlandesa (Monte Naranco): 3 *ex*. adults (A. Arias *leg*. and 1. *ex*. MNCN 15.05/98402 + 2. *ex*. OSF *deposit*.), 09‐VII‐2024, close to an urban fountain in a suburban area (30T 269,527 4,807,154) Hutchinson et al. 2022. Oviedo: surroundings of «Plaza Longoria Carbajal»: 2. *ex*. adults (A. Arias *leg*. and 1. *ex*. MNCN 15.05/98401 + 1. *ex*. OSF *deposit*.), 07‐X‐2024, gardened urban area (30T 269,206 4,805,247); Oviedo: gardens of the «El Cristo» Medical Center: 2. *ex*. adults (O. Sánchez *leg*. and OSF *deposit*.), 7‐X‐2024, gardened urban area (30T 268,073, 4,804,344); Oviedo: Montecerrao: 1. *ex*. adults (O. Sánchez *leg*. and OSF *deposit*.), 29‐X‐2024, gardened urban area (30T 268,300 4,803,916).

**FIGURE 2:**
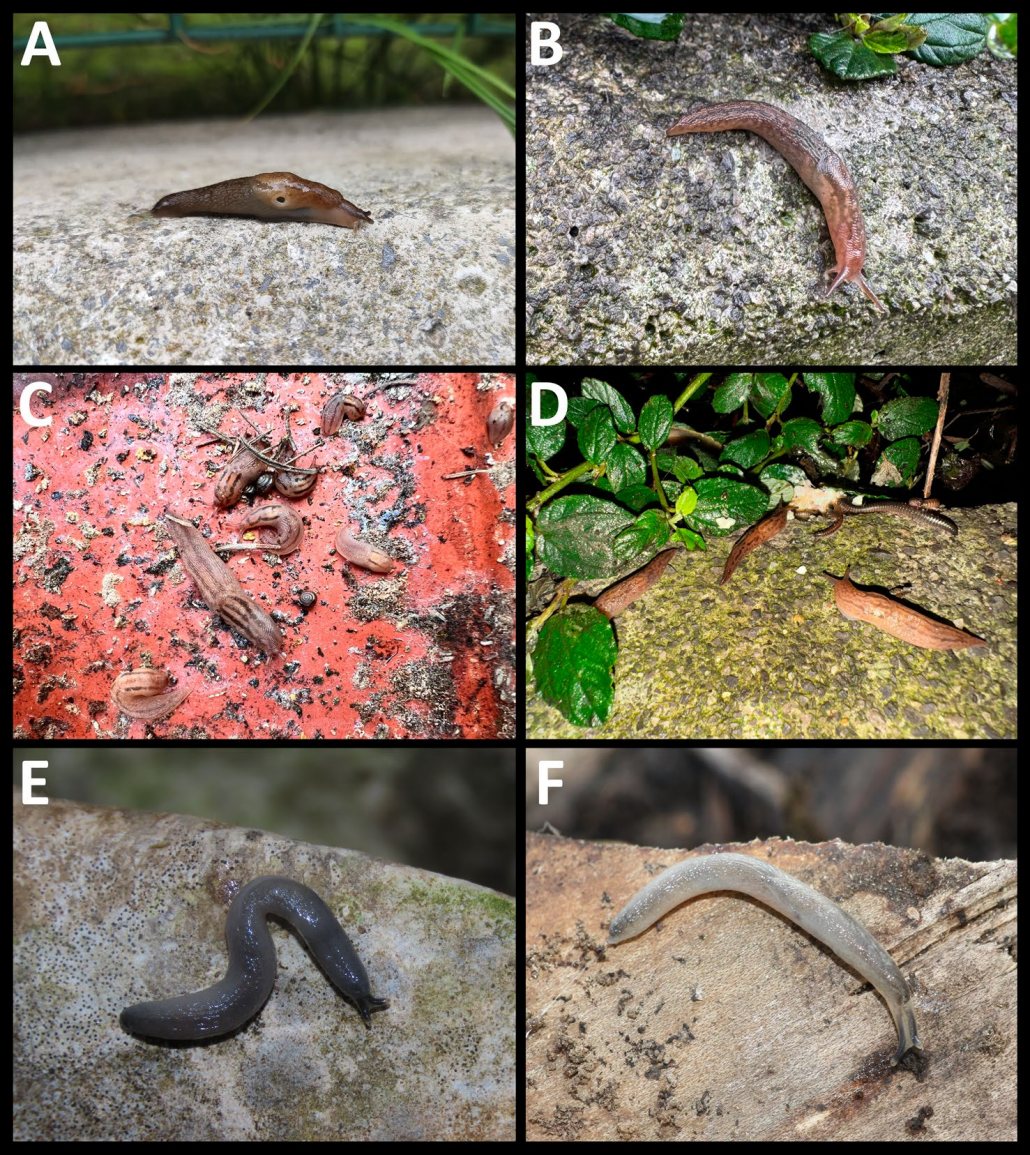
(A) Live specimens of *Ambigolimax parvipenis* from the garden of the «El Cristo» medical center (Oviedo) and (B–D) from Gran Bulevar ‘El Vasco’ shopping center (Oviedo), alone (B) or forming aggregations (C, D). (E) Alive juvenile specimens of *Boettgerilla pallens* from the Atlantic botanical garden (Gijón) and (F) Montecerrao (Oviedo).

**FIGURE 3:**
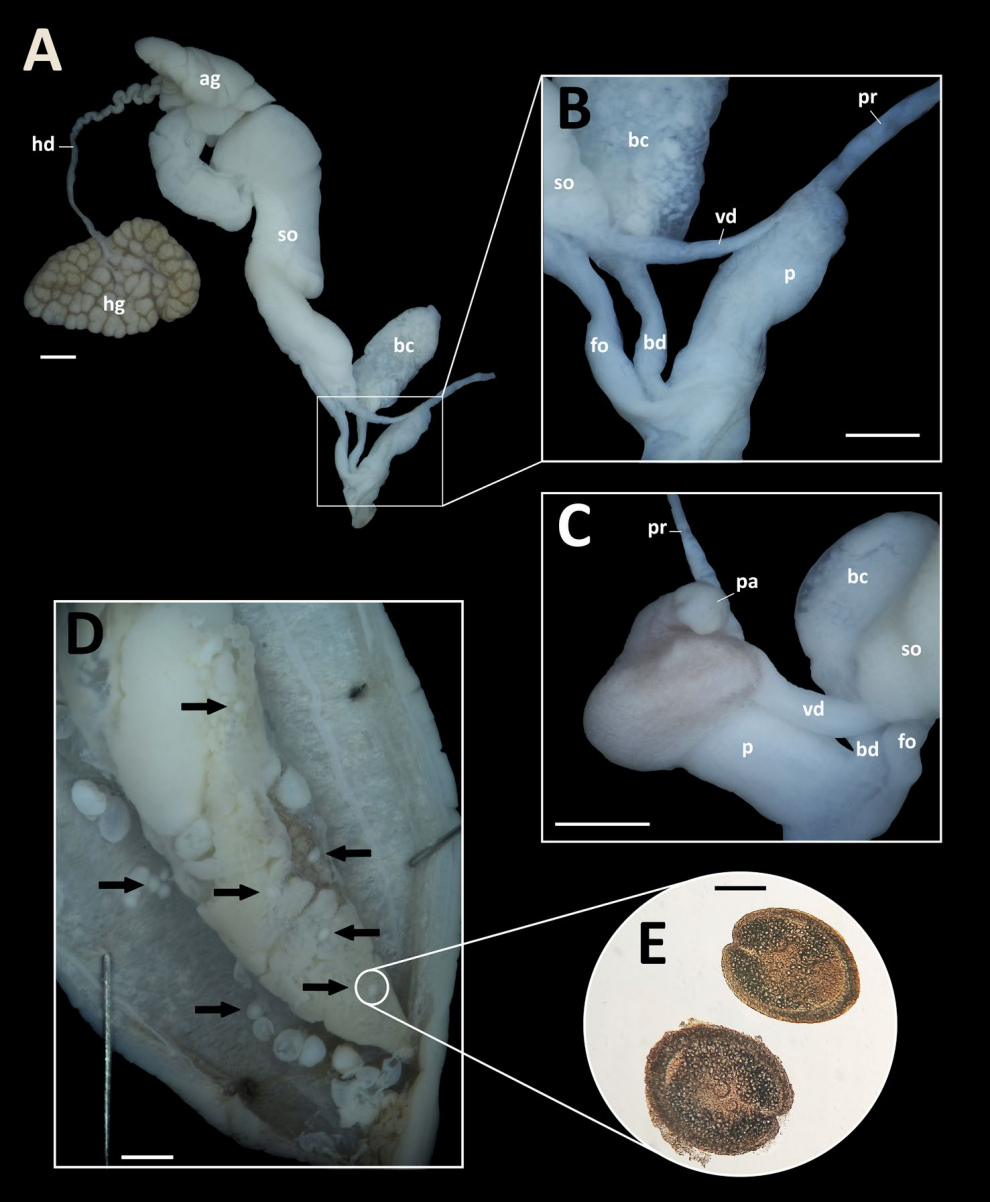
(A) Dissected genital tract of *Ambigolimax parvipenis*. (B) Detail of the male part of the genitalia of an *A. parvipenis* and (C) *A. valentianus*. (D) Internal part of a dissected *A. parvipenis* infected with cercariae (black arrows). (E) Microscopic detail of two cercariae. Ag = albumen gland; bc = bursa copulatrix; bd = bursa duct; fo = free oviduct; hd = hermaphroditic duct; hg = hermaphroditic gland; *p* = penis; pa = penial appendage; pr = penis retractor muscle; so = spermoviduct; vd = vas deferens. Scale bars A‐F: 1 mm; E: 0.1 mm.

Brief description. Medium‐sized slug with a slim body and a pointed tail that is softly keeled at the tip (Figure 2A–D). The sole is uniformly pale cream, and the tentacles are a dirty brown (Figure 2A–D). The mantle, which ranges from pale yellowish grey to light brown or dirty brown, covers one‐third of the body length, with the pneumostome located on its posterior half (Figure 2A). Most specimens exhibit a pair of thin, sharply defined dark stripes running along the middle of the back (Figure 2C). However, these stripes can sometimes be absent, wider and less distinct, or appear as irregular spots (Figure 2B,D). On the mantle, there is another pair of bands, which are almost always present, often accompanied by dark patches and fine dots (Figure 2B,D). Internally, the species is characterized by a short, unusually small penis without appendages. The absence of this appendage on the penis (Figure 3A,B) clearly differentiates it from *A. valentianus* (Férussac, 1821), the most similar species, which presents an appendage near the junction of the vas deferens and the penis retractor muscle (Figure 3C).

**FIGURE 4:**
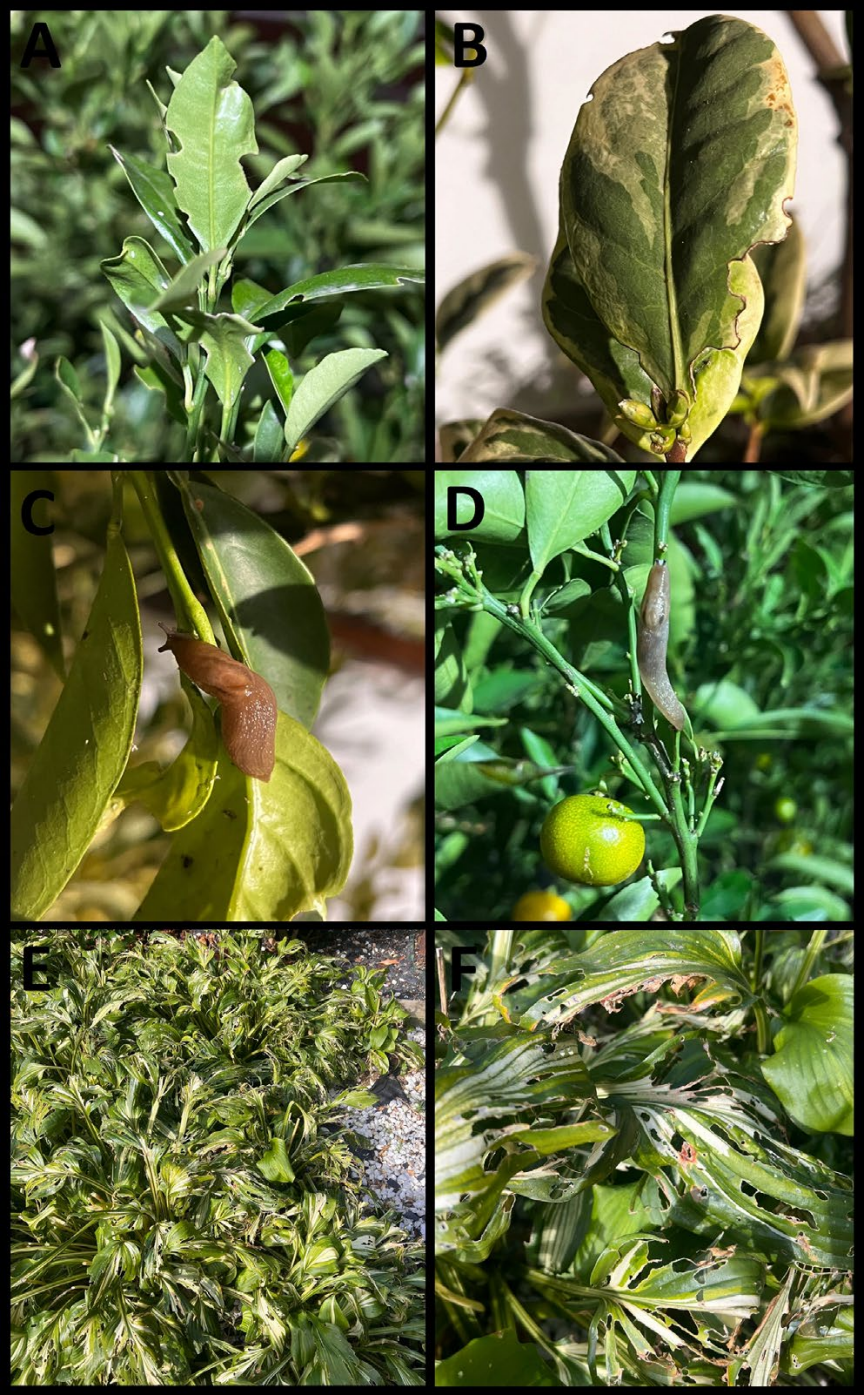
(A, B) Damaged leaves of sweet orange by *Ambigolimax parvipenis* and other slugs (C, D). *A. parvipenis* specimens feeding on sweet orange plants (E, F). Damaged leaves of the variegated plantain lily (*Hosta* spp.) by *A. parvipenis* and other slugs (E, F).

Remarks. Several adults, subadults and early juvenile stages were found coexisting in the same location throughout the year (Figure 2C), though specimens were only collected on specific dates. They were discovered in sympatry with the closest native species *A. valentianus*, at all sampled locations except for the gardens of the «El Cristo» Medical Center. *Ambigolimax valentianus* is externally identical to *A. parvipenis* in both size and colour polymorphism. Accurate identification between these species can only be confirmed through internal morphological (genitalia) (Figure 3B,C) and genetic analysis. These are the first confirmed records for Asturias and the first morphological confirmation for continental Spain. Hutchinson et al. (2022) suggested that the length rectal caecum as a useful character to distinguish *A. parvipenis* (stopping shorter than the tip of the visceral sac) from *A. valentianus* (almost reaches the tip or occasionally surpasses the visceral sac), but this proved not to be consistent among our specimens as already indicated by Turóci et al. (2023). After dissecting a specimen of *A. parvipenis* (16‐X‐2024, from The Gran Bulevar ‘El Vasco’), several cercariae of flatworms (Plathyelminthes: Trematoda: Digenea) were found infecting different parts of the internal anatomy of the animal (muscle, hepatopancreas and genitalia) (Figure 3D,E). The taxonomic identification of these flatworms cannot be carried out. Furthermore, several individuals located in ‘Plaza Longoria Carbajal’ (Oviedo, 16‐X‐2024) were observed feeding directly on different ornamental plants, such as the variegated sweet orange (*Citrus japonica* Thunb.) (Figure 4A–D) and the variegated plantain lily (*Hosta* Tratt. spp.) (Figure 4E,F). External damages to the plants were seen mostly in the leaves. Although all the individuals collected while feeding on these plants corresponded to *A. parvipenis*, we cannot rule out that the damage observed was only caused by this species. Furthermore, specimens feeding on animal debris, such as dead slugs and snails and dog faeces, were also found.

Habitat. All specimens were found within the urban matrix of Oviedo, except for the peri‐urban locality of Pista Finlandesa. Natural vegetation in these gardened areas was scarce and barely represented by some opportunistic species, such as *Potentilla reptans* L. or *Senecio vulgaris* L. However, alien plants (e.g., *Oxalis corniculata* L., *Veronica persica* Poir.) find a place to grow in these altered habitats, mainly due to the constant mowing, trampling, irrigation and perturbations these areas are subjected to. A common practice is to partially cover these areas with pebbles, timber, or wood pellets, which are exploited as shelter by both native and alien fauna. Additionally, many exotic species are grown on purpose in these gardens for ornamental reasons: *Sarcococca orientalis* C.Y.Wu and *Ligustrum ovalifolium* Hassk. from Asia or *Ceanothus thyrsiflorus* Eschw. from North America as ornamental bushes or *Syagrus romanzoffiana* (Cham.) Glassman from South America as ornamental palm. Nevertheless, some native plants are occasionally used in gardening (e.g., *Laurus nobilis* L. is often used in fringes) but are surely grown along exotic alien plants that act as vectors for the introduction of exotic alien animals and fungi. Regarding Pista Finlandesa, several buildings and particular gardens occur there, and it is not uncommon to see pruning and mowing remains, which could be the source of the entrance of new exotic species.

Accompanying mollusc species. *Ambigolimax parvipenis* was found at Gran Bulevar ‘El Vasco’ shopping center coexisting with other species of slugs including *A. valentianus*, the leopard slug *Limax maximus* , the yellow slug *Limacus flavus* (Linnaeus, 1758), the shelled slug *Testacella maugei* Férussac, 1819, and several specimens of an unidentified species of the genus *Arion*. They have also been found together with the exotic snail *Zonitoides arboreus* (Say, 1817) and the native ones, *Cornu aspersum* (O. F. Müller, 1774) and *Oxychilus draparnaudi* (H. Beck, 1837). In the locality of Montecerrao, it was found coexisting with *B. pallens* , *Deroceras reticulatum* (O. F. Müller, 1774), and *Deroceras* sp. In the remaining localities, it has only been seen together with *A. valentianus*.

Family Boettgerillidae Wiktor & I. M. Likharev, 1979.

Genus *Boettgerilla* (Simroth 1910).

*Boettgerilla pallens* (Simroth 1912).

Material examined. SPAIN: Principality of Asturias: Gijón: Atlantic Botanical Garden: ‘Cantabrian forests area’: 2 *ex*. subadult (O. Sánchez *leg*. and OSF *deposit*.), 04‐III‐2024, gardened area of an urban botanical garden (30T 288,649 4,822,014); Atlantic Botanical Garden: ‘Endemic, rare and threatened plants area’: 4 *ex*. subadult (O. Sánchez *leg*. and OSF *deposit*.), 06‐V‐2024, gardened area of an urban botanical garden (30T 288,558 4,822,049) Simroth 1912. Oviedo: Montecerrao: 3. *ex*. subadult (O. Sánchez *leg*. and 2. *ex*. MNCN 15.05/98404 + 1. *ex*. OSF *deposit*.), 29‐X‐2024, gardened urban area (30T 268,300 4,803,916).

Brief description. The worm slug *Boettgerilla pallens* is a small to medium‐sized slug with a slender, distinctly wormlike body (Figure 2E,F). The tail is pointed and strongly keeled. Adult specimens are typically lead‐grey in colour, fading to almost white in front of the mantle, along the lower flanks and on the sole, with a darker head and tail. Juveniles are paler (Figure 2E,F). The pneumostome, slightly paler than the mantle in adults, is located just over halfway along the mantle. *B. pallens* has a thin internal shell, usually with an irregular outline.

Remarks. Only nine subadult specimens were collected at both localities. Studying the genitalia was not possible, as they were not completely developed. However, the external characteristics clearly distinguish this species. The genitalia can be examined in Simroth (1912), and in Balashov and Baidashnikov (2012). Juveniles may be misidentified as *Deroceras* juveniles due to their pale appearance. However, the strong keel at the end of the tail provides a reliable distinguishing feature. This is the first confirmed record for Asturias and continental Spain.

Habitat. Specimens from the Atlantic Botanical Garden were found non‐active under stones and among the leaf litter during daytime samples. Some specimens were in the ‘Cantabrian forests’ section, mainly artificially recreated forests and woodlands from the Cantabrian region, especially of dominated by broadleaved deciduous trees, such as *Quercus robur* L. or *Fagus sylvatica* L. Other specimens were in the section ‘Endemic, rare and threatened plants’, a garden area with several grass species such as *Poa annua* L. or *Veronica persica* Poir., that grow naturally there. Both areas are meant to represent natural habitats, species and environmental conditions from the Cantabrian region. However, exotic plants are used in the surroundings of these sections, especially in the ‘La Isla’ historical garden, which is a part of the botanical garden mainly focused on ornamental plants and adjacent to both sections. Respecting the Montecerrao locality, it would be found in the peri‐urban area of Oviedo and, thus, subjected to the same treatments and disturbances as the previously mentioned garden areas where the *A. parvipenis* was found.

Accompanying mollusc species. *Boettgerilla pallens* was found at Atlantic Botanic Garden of Gijón coexisting with other species of slugs including the leopard slug *Li. maximus*, in the Cantabrian forests area and with several snail species like *Cornu aspersum*, *Cepaea nemoralis* (Linnaeus, 1758), *Cochlicopa lubricella* (Porro, 1838), *Vallonia costata* (O. F. Müller, 1774), *Lauria cylindracea* (Da Costa, 1778), *Perpolita hammonis* (Strøm, 1765) and *Cochlicella barbara* (Linnaeus, 1758). In the locality of Montecerrao, it was found coexisting with *A. parvipenis*, *Deroceras reticulatum* (O. F. Müller, 1774), and *Deroceras* sp.

### 3.2 Genetic Results

**FIGURE 5:**
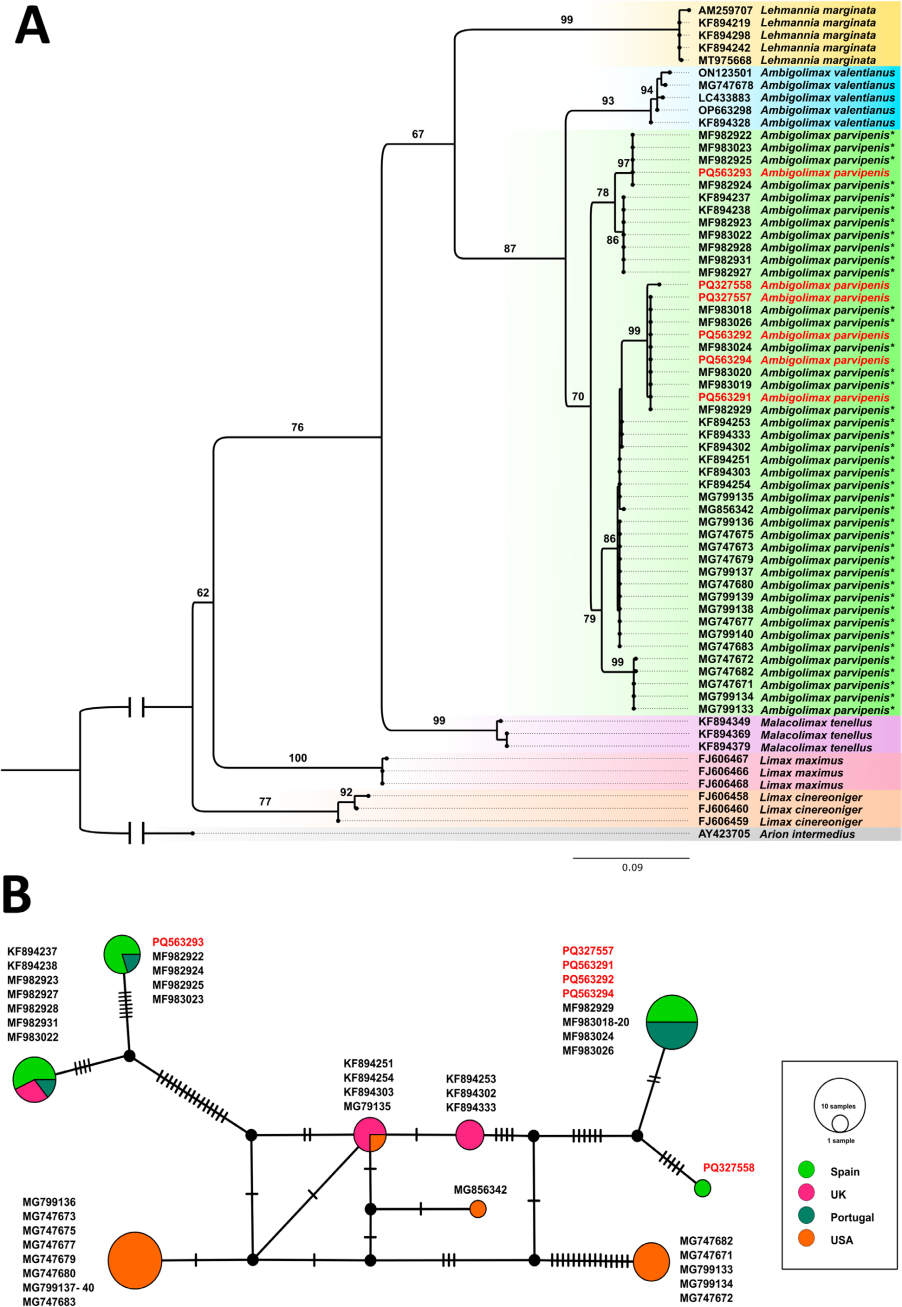
(A) Maximum likelihood phylogeny inferred using the COI dataset in IQ‐TREE for *Ambigolimax parvipenis* samples. Ultrafast bootstrap values are provided at relevant nodes. GenBank accession numbers are listed adjacent to each scientific name. The new sequences are highlighted in red. Each colour represents a different species. Names marked with (*) refer to names that have been updated according to Hutchinson et al. (2022). (B) The mitochondrial haplotype COI network from available sequences of *Ambigolimax parvipenis*. The legend shows the country where *Ambigolimax parvipenis* samples were sequenced (Genbank accession numbers and precise locations in Appendix S1). Node sizes are proportional to the number of sequences in which the haplotype was observed. Bars indicate the number of mutations needed to get from one haplotype to another, and black circles represent hypothetical nodes.

The Blast identification engine identified the six sequences of *A. parvipenis* specimens with 94.96%–98.93% pairwise identity with 16 sequences of *Lehmannia nyctelia* and *Lehmannia* spp. corrected by Hutchinson et al. (2022) as *A. parvipenis* (see Discussion). The remaining hits were with other species of the genus *Lehmannia* and *Ambigolimax* with a similarity of 92.6% or less. The phylogenetic analysis revealed that samples assigned to *A. parvipenis* based on morphology formed a well‐supported group with a high bootstrap support (i.e., 70%) (Figure 5A). The COI sequence divergence for *A. parvipenis* appears to be very high, in some cases reaching 5% between sequences. The haplotype analyses using PopART revealed a high haplotypic diversity among the available sequences of the species (*n* = 41) and among those here analysed (*n* = 6). The samples analysed were represented by three haplotypes. The first one groups the sequences from the Centro Medico del Cristo (PQ563291–PQ563292) as well as two samples from Gran Bulevard El Vasco (PQ327557, PQ563294) with the available sequences from Serra de Monchique (MF983024, MF983026) and several sequences from Serra da Estrela and Deleitosa (MF982929, MF983018–MF983020). The second haplotype groups the sequence from the Plaza Longoria Carbajal (PQ563293) with several sequences from Deleitosa (MF982922, MF982924–MF982925) and Serra da Estrela (MF983023). Finally, a specimen from Gran Bulevard El Vasco presented a haplotype not shared by any available sequence of the species (Figure 5B; Appendix S1).

**FIGURE 6:**
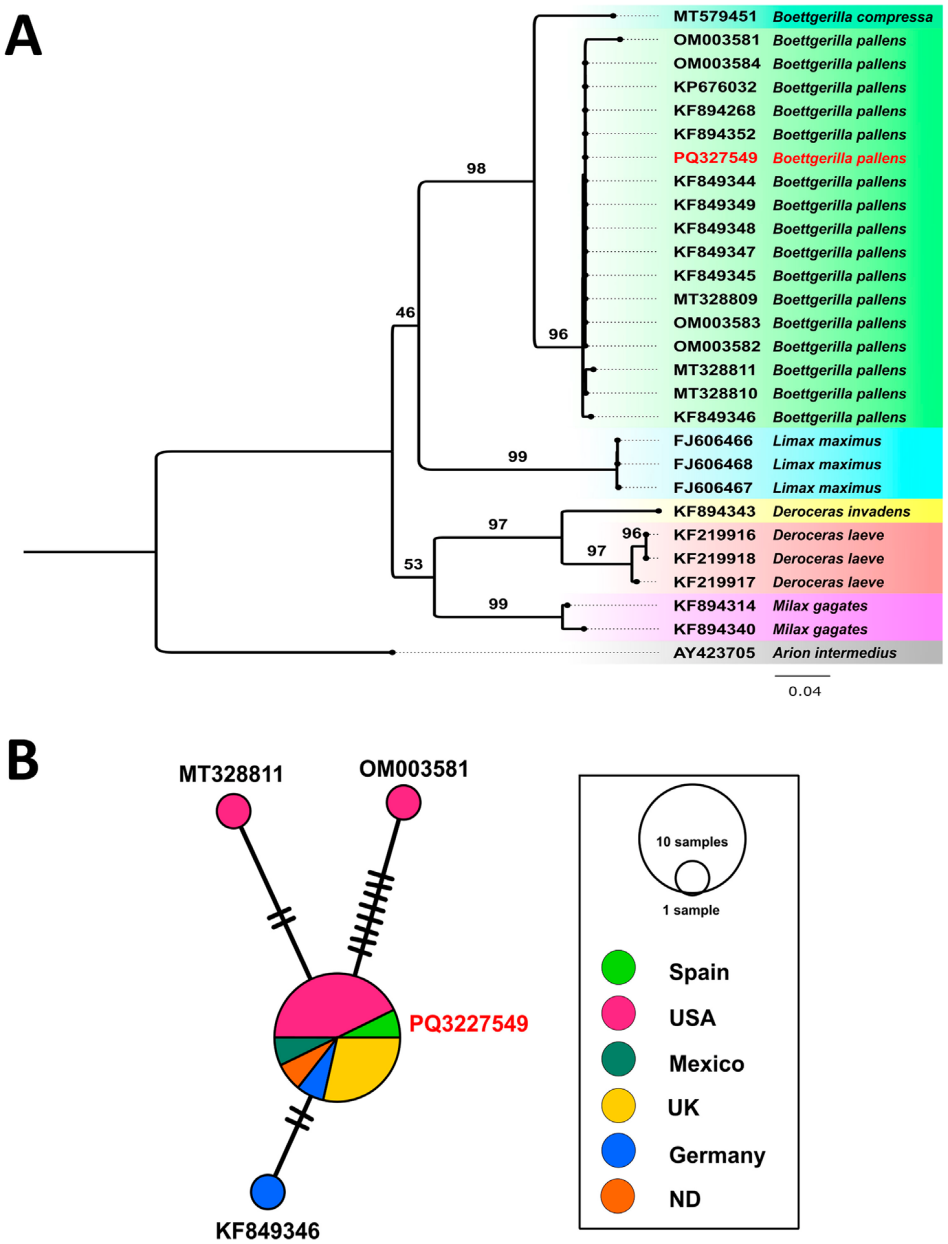
(A) Maximum likelihood phylogeny inferred using the COI dataset in IQ‐TREE for *Boettgerilla pallens*. Ultrafast bootstrap values are provided at relevant nodes. GenBank accession numbers are listed adjacent to each scientific name. The new sequence is highlighted in red. Each colour represents a different species (B). The mitochondrial haplotypes COI network from available sequences of *Boettgerilla pallens*. The legend shows the country where *Boettgerilla pallens* samples were sequenced (GenBank accession numbers and precise locations in Appendix S1). Node sizes are proportional to the number of sequences in which the haplotype was observed. Bars indicate the number of mutations needed to get from one haplotype to another. ND: No locality data available.

On the other hand, the Blast identification engine identified the sequence of *B. pallens* with 97.52%–100% pairwise identity with 12 sequences of *B. pallens* . The remaining hits were with other species of different terrestrial molluscs and slugs with a similarity equal to or less than 92.66%. The phylogenetic analysis revealed that our sample assigned to *B. pallens* formed a well‐supported group with a high bootstrap support (i.e., 96%) (Figure 6A). The haplotype analyses using PopART revealed low haplotypic diversity among the available sequences of the species (*n* = 12) (Figure 6B). Only four haplotypes are shown, separated by only a few mutations (i.e., 2–8) (Figure 6B). The individual studied has the most common haplotype (*n* = 9), shared with individuals from Europe (Germany and United Kingdom) and America (United States and Mexico) (Figure 6B; Appendix S1).

## 4 Discussion

Land slugs have well‐known histories of invasions throughout the world (Vendetti et al. 2018). Among them, some of the most interesting are the cases of *D. invadens* (Reise et al. 2011; Hutchinson et al. 2020) and *A. vulgaris* (Pfenninger et al. 2014; Zajak et al. 2017), or the ones here considered: *A. parvipenis* (Hutchinson et al. 2022; Hart et al. 2023) and *B. pallens* (Reise et al. 2000). Most of these invasion stories are characterized by the same features: (a) difficulty in differentiating native slugs from alien ones; (b) the impossibility of tracing their native and introduced distribution areas due to their old and extended silent invasion; and (c) the limited knowledge available about their ecology, morphology and potential impacts in invaded ecosystems. Therefore, any contribution is important to unravelling the invasion histories of these species. In this context, our work presents an important breakthrough on two of these species. One of the cases studied here is the slug known as *Ambigolimax parvipenis* (Hutchinson et al. 2022). *Ambigolimax parvipenis* has been recently described based on the revision of the historical material incorrectly identified as *Lehmannia nyctelia* (=*Letourneuxia nyctelia* (Bourguignat, 1861)) throughout the world (Rowson et al. 2014; Vendetti et al. 2018) and now is considered as «*An invasive species of unknown origin*» (Hutchinson et al. 2022; Hart et al. 2023). New molecular studies with specimens from new locations are necessary to unveil their native and introduced range. Hutchinson et al. (2022) indicated that its current known distribution comprises the Western United States, the British Isles and the westernmost mainland and island parts of Europe (France, Greece and Spain). Recently, it has been reported in Hungary (Turóci et al. 2023). Regarding the situation in the Iberian Peninsula, Hutchinson et al. (2022) do not examine new Iberian material in their work but attribute 15 GenBank sequences identified as *Lehmannia* sp. (Gómez‐Rodríguez et al. 2018) to *A. parvipenis* without specifying precise localities. We have been able to trace the sequences and know their precise location (C. Gómez‐Rodríguez pers. comm.; see them in Appendix S1). Eight correspond to Deleitosa (Cáceres, Spain), five to Serra da Estrela (Guarda, Portugal) and two to Serra de Monchique (Faro, Portugal). Thus, we have a previous molecular confirmation of presence in Portugal and Spain, and we have contributed with new locations in Northern Spain (Asturias) with the first morphological characterisation. This species was also recorded in several insular Spanish areas, such as the Canary and the Chafarinas Islands (Hutchinson et al. 2022). It is important to highlight the high degree of genetic diversity of the available sequences together with the newly presented ones here, with up to 5% genetic difference in the COI fragment. This may reflect a greater genetic plasticity of the species that is giving it some kind of advantage in the face of invasions, being able to deal easily with the stochastic events of genetic drift associated with introductions (Bélouard et al. 2019). On the other hand, the haplotypic study suggests a common origin of the known Iberian populations, as two of the three haplotypes found in Asturias correspond to the same haplotypes discovered in Gómez‐Rodríguez et al. (2018). Similarly, there seems to be a haplotype that links the populations reported by Gómez‐Rodríguez et al. (2018) with those from South Devon in the United Kingdom (Rowson et al. 2014).

Specimens reported by Gómez‐Rodríguez et al. (2018) and ours were found in anthropized habitats (urban or suburban areas), usually associated with gardened areas with ornamental vegetation. This seems to support the introduced origin of the species in the region through gardening, as a stowaway in pots, soil, or on the ornamental plants themselves, a way of introduction typical for slugs (Cowie et al. 2008). The impacts of *A. parvipenis* are still unknown and imprecise, but we can make several first inferences. This slug has a quite generalist feeding behavior and can affect ornamental plants in urban areas. This could be a problem in these gardened areas, as well as in nursery facilities, as it could behave like a pest species. Furthermore, another important risk is the presence of trematode cercariae in at least one examined specimen. Although it has not been possible to determine the species of the parasite, this may suggest two facts: (a) either it is a parasite that has managed to parasitise *A. parvipenis* but uses another native mollusc species as its usual host, or (b) it is a trematode that has been imported with this species from elsewhere and could have a negative impact on native species whether it infects them. There are some cases of infections by trematodes in other closest species of the genus *Ambigolimax* (e.g., Ivanova et al. 2019) and a health risk for native fauna, livestock and humans cannot be ruled out (Sánchez et al. 2021).

Another case of silent invasion is *Boettgerilla pallens* (Reise et al. 2000), first described in the montane forests of the Caucasus (Abkhazia and Georgia), alongside the only other species in its family, *B. compressa* (Simroth 1910). Since then, *B. pallens* has expanded across the Northern Hemisphere, spreading throughout Europe (Reise et al. 2000; Balashov and Baidashnikov 2012; Rowson et al. 2014; Margry 2014), Central Asia (Schikov 2017), and America (Hausdorf 2002; Araiza‐Gómez et al. 2015; McDonnell et al. 2014, 2020; Dodge et al. 2022). Several studies from the United Kingdom, Belgium and Austria report its rapid spread within a few years, often coinciding with intensified sampling efforts (see Reise et al. 2000), a trend also currently observed in North America (Reise et al. 2000; McDonnell et al. 2014, 2020; Catling and Kostiuk 2018; Dodge et al. 2022). This species is primarily found in anthropogenic or semi‐natural habitats such as parks, gardens, nurseries, greenhouses and botanical gardens (Reise et al. 2000; Grimm et al. 2009; McDonnell et al. 2020). The movement of plants for horticulture likely facilitated its spread (Reise et al. 2000; Catling and Kostiuk 2018). Our records from a public botanical garden and urban gardened areas align with previous cases, where such sites remain focal points for the expansion of this species (Reise et al. 2000; McDonnell et al. 2020). Regarding the Iberian distribution, Borredà et al. (1994) collected in 1991 one single specimen in Andorra. However, it is likely that *B. pallens* had been established for some time in other locations but went unnoticed due to its cryptic nature. Despite being an introduced species, its invasive status and economic significance remain debated (McDonnell et al. 2020). This species has subterranean habits (Seidl and Seidl 1997; Reise et al. 2000), feeding on earthworm faeces, carrion, roots, decaying vegetation, detritus and eggs of various gastropod species (Gunn 1992; Forsyth 2004; Rowson et al. 2014), and it has occasionally been reported as a pest of ornamental plants (Godan 1983) and potatoes (Moolenbeek 2002). Additionally, its impacts may be overshadowed by the more common presence of other slugs that are significant horticultural and agricultural pests (Reise et al. 2000; McDonnell et al. 2020). However, synergistic impacts with other molluscs as a plant pest (McDonnell et al. 2020) or potential competition with native fauna cannot be ruled out. Further studies are needed to determine not only its distribution but also whether it has any significant impacts. Molecular data for this species are scarce and show a low haplotypic diversity, with the analysed population sharing the same haplotype as certain specimens from Europe (Germany and the United Kingdom) and America (the United States and Mexico), proposing a common origin of these populations.

## 5 Conclusions

Invasive species pose a significant threat to biodiversity and ecosystem stability. This study provides the first confirmed morphological records of *Ambigolimax parvipenis* and *Boettgerilla pallens* in mainland Spain, expanding the known distribution of these taxa. The findings highlight the role of urban and peri‐urban gardened areas as introduction hubs for non‐native gastropods, likely facilitated by the trade and movement of ornamental plants. The integration of morphological and genetic analyses enabled accurate species identification, addressing taxonomic challenges associated with cryptic invasions. Additionally, the detection of trematode infections in *A. parvipenis* raises concerns regarding potential parasitic transmission and ecological interactions with native fauna. These results underscore the necessity of continuous monitoring and early detection of exotic species to prevent their silent spread and mitigate potential ecological and economic impacts. Further research is required to assess the long‐term establishment, dispersal mechanisms and ecological implications of these introduced gastropods.

## Author Contributions

**Omar Sánchez:** conceptualization (equal), formal analysis (lead), investigation (equal), methodology (equal), resources (equal), writing – original draft (lead), writing – review and editing (lead). **Víctor González‐García:** investigation (equal), resources (equal), writing – review and editing (equal). **Jairo Robla:** formal analysis (equal), investigation (equal), writing – review and editing (equal). **Andrés Arias:** conceptualization (lead), funding acquisition (equal), investigation (equal), resources (equal), supervision (lead), writing – original draft (equal), writing – review and editing (lead).

## Conflicts of Interest

The authors declare no conflicts of interest.

## Supporting information


Appendix S1.

## Data Availability

All the genetic data are available at GenBank using the accession numbers provided in Figures 5 and 6 and in Appendix S1.
